# Crystal structure of a Zn complex with tereph­thalate and 1,6-bis­(1,2,4-triazol-1-yl)hexa­ne

**DOI:** 10.1107/S2056989017017224

**Published:** 2018-01-01

**Authors:** Taisiya S. Sukhikh, Evgeny Yu. Semitut, Andrei S. Potapov

**Affiliations:** a Nikolaev Institute of Inorganic Chemistry, SB Russian Academy of Sciences, Akad. Lavrentiev prospekt 3, Novosibirsk 90, 630090 , Russian Federation; b Department of Natural Sciences, National Research University, Novosibirsk State University, Pirogova st. 2, Novosibirsk 90, 630090 , Russian Federation; c Department of Biotechnology and Organic Chemistry, National Research Tomsk Polytechnic, University, 30 Lenin Ave., 634050, Tomsk, Russian Federation

**Keywords:** crystal structure, flexible ligand, terephthalate, coordination polymer

## Abstract

A new zinc coordination polymer with rigid benzene-1,4-di­carboxyl­ate (bdc) and flexible 1,6-bis­(1,2,4-triazol-1-yl)hexane (btrh), namely poly[[(μ_2_-benzene-1,4-di­carboxyl­ato)[μ_2_-1,6-bis­(1,2,4-triazol-1-yl)hexa­ne]zinc] di­methyl­form­amide monosolvate], [Zn(C_8_H_4_O_4_)(C_10_H_16_N_6_)]·C_3_H_7_NO, was synthesized. According to the single-crystal XRD analysis, the product crystallizes in the *P*-1 space group and has a layered structure. Analysis of the layered structure reveals {Zn(bdc)} chains which are connected by pairs of btrh ligands. The layers are packed tightly perpendicular to the [1-22] direction, separated by one non-disordered di­methyl­formamide solvent mol­ecule per formula unit. According to thermogravimetric analysis, the product completely loses this solvent at 453 K; the desolvated compound is stable up to 503 K. As a result of the lack of hydrogen-donor groups, hydrogen bonds are not observed in the structure of the complex; however, an inter­molecular C—H⋯π contact of 3.07 Å occurs.

## Abstract 2

A new Zn coordination polymer with bitopic rigid terephthalate and flexible 1,6-bis­(1,2,4-triazol-1-yl)hexane was synthesized and structurally characterized.

## Chemical context   

Coordination polymers with flexible bitopic ligands have attracted great inter­est as prospective materials for gas separation, sensing materials, electrochemical devices or catalysis (Pettinari *et al.*, 2016 ▸). One of the favoured classes of bitopic ligands are bis­(azol-1-yl) alkanes, which have been used for the preparation of various transition metals coordin­ation polymers with different topologies (Alkorta *et al.*, 2017 ▸; Pellei *et al.*, 2017 ▸; Manzano *et al.*, 2016 ▸; Liu *et al.*, 2012 ▸). Bitopic bis­(azol-1-yl)alkanes have two separated metal-binding sites that allow them to form a wide variety of polymeric structures. Thus, coordination compounds based on these ligands could be applied in the design of various functional materials with a wide range of potential applications. Recently, we have synthesized three new Zn coordination polymers based on bis­(triazol-1-yl)propane and terephtalate anions (Semitut *et al.*, 2017 ▸). By varying the conditions, it was possible to synthesize three different polymeric compounds, which have inter­esting luminescent properties. As part of our studies with the aim of preparing new coordination polymers with flexible bis­(triazol-1-yl)alkane ligands, we report herein the synthesis and crystal structure of [Zn(bdc)(btrh)]·DMF (bdc = benzene-1,4-di­carboxyl­ate, btrh = 1,6-bis­(1,2,4-triazol-1-yl)hexane, DMF = di­methyl­formamide).
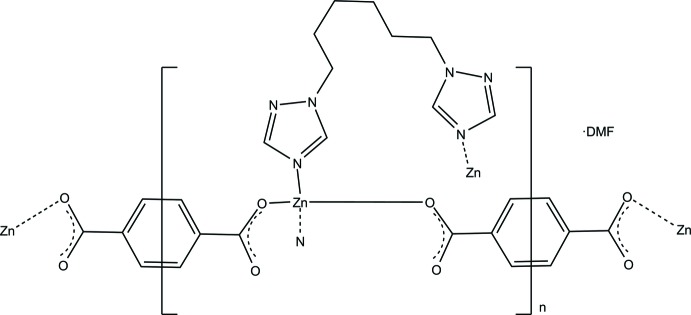


**Figure 1:**
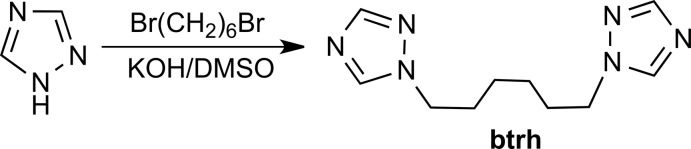
Synthesis of 1,6-bis­(1,2,4-triazol-1-yl)hexane.

The btrh ligand (Fig. 1 ▸) was prepared by the reaction of 1,2,4-triazole with 1,6-di­bromo­hexane in a superbasic dimeth­yl sulfoxide–potassium hydroxide medium using our modified procedure reported for bis­(triazol­yl)propane (Semitut *et al.*, 2017 ▸). Our proposed procedure does not require the use of toxic solvents and gives higher yields compared to the literature procedure (Liu *et al.*, 2012 ▸). The title complex was prepared by the reaction of zinc nitrate, btrh and terephthalic acid under solvothermal conditions (368 K) in DMF. The product was formed after 48 h as a crystalline colourless solid of plate-like shape. The single crystal used for structure determination was collected from the filtered product. The polycrystalline compound was characterized by elemental (C, H, N) and powder XRD analysis (Fig. S1, Supporting information), indicating formation of this complex as a main phase.

## Thermal stability   

**Figure 2:**
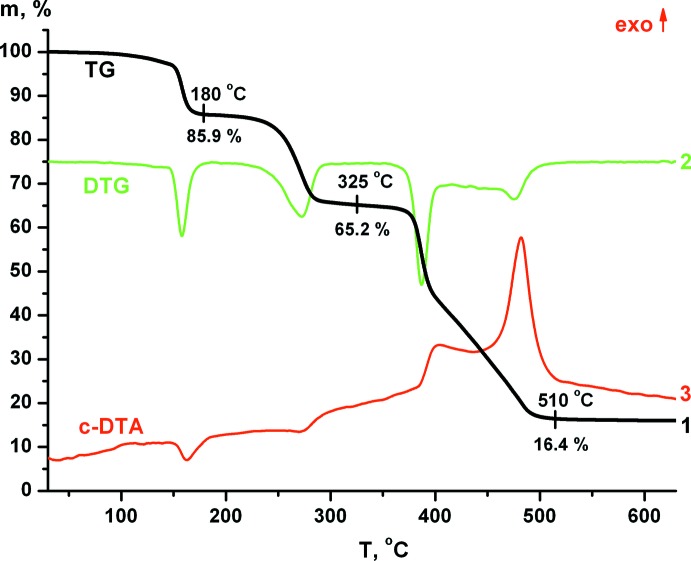
Curves of thermal analysis for [Zn(btrh)(bdc)]·DMF in O_2_/Ar (21%) atmosphere; 1 TG, 2 DTG, 3 c-DTA.

The thermal stability of the synthesized coordination polymer was studied in oxidative O_2_/Ar (21%) atmosphere. Thermogravimetric measurements were carried out on a NETZSCH thermobalance TG 209 F1 Iris. Open Al_2_O_3_ crucibles were used (loads 7–10 mg, heating rate 10 K min^−1^). The thermal analysis of [Zn(btrh)(bdc)]·*n*DMF revealed that the synthesized compound has three thermolysis stages in an oxidative atmosphere (Fig. 2 ▸). The first stage of thermolysis is the process of the loss of solvate mol­ecules that runs in the range of 373–453 K and has a well-defined step on the TG curve. The mass loss of solvate mol­ecules corresponds to a composition with *n* ≃ 1, which is in good agreement with the crystal data. The desolvated compound is stable up to 503 K. The second and third stages run in the ranges 503–573 and 633–773 K, respectively. The second stage corresponds to partial degradation of btrh and terephtalate and third to further decomposition and the burning process of the formed carbon products, resulting in the formation of ZnO according to powder XRD analysis.

## Structural commentary   

**Figure 3:**
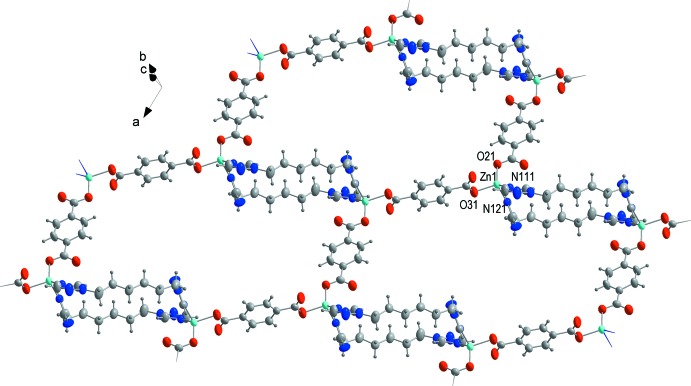
Displacement ellipsoid plot of a single layer of the coordination polymer showing ellispoids drawn at the 50% probability level.

The structure is a 2D coordination polymer crystallizing in space group *P* 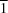. The central Zn atom has a distorted tetra­hedral environment comprising two oxygen and two nitro­gen atoms. It is coordinated by two crystallographically independent (bdc)^2−^ ligands (halves), forming zigzag chains along the [210] direction, which are linked by btrh ligands (Fig. 3 ▸). Contrary to our recently reported Zn complexes with 1,3-bis­(1,2,4-triazol-1-yl)propane containing a shorter alkyl bridge (Semitut *et al.*, 2017 ▸), 1,3-bis­(pyrazol-1-yl)propane (Potapov *et al.*, 2012 ▸) and bis­(imidazol-1-yl)alkanes (Barsukova, Samsonenko *et al.*, 2016 ▸; Barsukova, Goncharova *et al.*, 2016 ▸), the title compound is a 2D polymer, because the Zn atoms are connected by btrh ligands in pairs, not in chains, thus preventing the formation of a 3D net. Each Zn atom is linked with three others *via* (1) the first bdc^2–^ ligand, (2) a second bdc^2–^ ligand and (3) a pair of btrh ligands. The layers of the title compound are arranged perpendicular to the [1
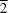
2] direction in such a way that the {Zn_2_(btrh)_2_} units lie between the hollows of neighboring layers (Figs. S2, S3).

## Supra­molecular features   

Layers of the complex are packed tightly, revealing only one DMF solvent mol­ecule per formula unit. Analysis of the residual electron-density map clearly indicates the presence of a not or very slightly disordered DMF mol­ecule (Fig. S4). After refining DMF, only one peak of 0.60 e Å^−3^ (attributed to a C atom of occupancy *ca* 0.15) is observed, while the densities of other peaks coincide with those of holes (*ca* ±0.3 e Å^−3^). Thus, the DMF mol­ecule is rather not disordered. Besides disorder, atomic displacement parameters that are larger than those for other atoms can be due to partial loss of the solvent during the experiment. DMF mol­ecules are located in the channel voids, which occupy 26.4% of the structure (Fig. S5). As a result of the lack of H-donor groups, hydrogen bonds are not observed in the structure of the complex; however, inter­molecular C—H⋯π contacts of 3.07 Å (Table 1 ▸) occur between the aromatic rings of bdc ligands (Fig. S6). These contacts connect neighbouring layers.

## Database survey   

A database survey showed that the majority of the known structures of polymers with flexible bis­(azol-1-yl)alkanes are compounds based on relatively short linkers (from methane to penta­ne) but that the number of polymers based on longer linkers (having a CH_2_-chain higher than six) is relatively low. The lack of structural information on long flexible ligands can be due to the fact that it is more difficult to obtain single crystals of good quality for these compounds. Such ligands tend to form inter­penetrated polymers with disorder and a variety of modifications. A search of the Cambridge Structural Database (CSD, Version 5.38, update May 2017; Groom *et al.*, 2016 ▸) for compounds containing btrh and any metal gave 51 hits, of which only one contains both btrh and bdc ligands (refcode ETAKAM; Zhang *et al.*, 2011 ▸). This Cd polymer also has a 2D structure, but the {Cd(bdc)} chains are linear and are inter­sected by {Cd(btrh)} chains. Thus, contrary to our case, the two central metal atoms are connected by only one btrh ligand.

## Synthesis and crystallization   

**Starting materials and experimental procedures**

The starting reagents used for the synthesis of the coordination compound – Zn(NO_3_)_2_·6H_2_O (chemical grade), dimethyl formamide (analytical grade) and terephthalic acid (analytical grade) – were used as received.

NMR spectra were recorded on a Bruker AV300 instrument operating at 300 MHz for ^1^H and 75 MHz for ^13^C, solvent residual peaks were used as inter­nal standard. Elemental analyses were carried out on a Eurovector EuroEA 3000 analyser. Infrared (IR) spectra of solid samples as KBr pellets were recorded on a FT-801 spectrometer (4000–550 cm^−1^). The powder XRD data were collected with a DRON RM4 powder diffractometer equipped with a Cu *K*α source (λ = 1.5418 Å) and graphite monochromator for the diffracted beam.

**Synthesis of compound [Zn(btrh)(bdc)]·** ***n*** **DMF**

35.2 mg (0.16 mmol) of btrh ligand and 4.0 ml of Zn(NO_3_)_2_·6H_2_O (0.04 *M*) were added to 0.4 ml of a DMF solution of H_2_bdc (0.4 *M*) in a glass vial. The resulting mixture was stirred for several minutes at room temperature for total ligand dissolution and placed into an oven at 368 K. After heating for 48 h, the vial was cooled to room temperature. Plate-like colourless crystals formed on the bottom of the vial; they where filtered and washed twice with 5 ml of DMF and dried in a vacuum. The yield was 39 mg (53%). IR bands, cm^−1^: 3115, 2948, 2861, 1680, 1611, 1530, 1499, 1437, 1391, 1345, 1287, 1217, 1136, 1098, 1017, 1001, 947, 905, 878, 828, 750, 743, 673, 642, 577. Elemental analysis: found, %: C 48.5, H 5.9, N 18.9; calculated ([Zn(btrh)(bdc)]·DMF), %: C 48.2, H 5.2, N 18.8.

**Synthesis of 1,6-bis­(1,2,4-triazol-1-yl)hexane (btrh)**

A suspension of 2.76 g (40 mmol) of 1,2,4-triazole and 4.48 g (80 mmol) of powdered KOH in 15 ml of DMSO was stirred vigorously at 353 K for 30 min. The reaction flask was then immersed into a cold water bath and, after cooling to room temperature, 4.88 g (20 mmol) of 1,6-di­bromo­hexane in 10 ml of DMSO were added dropwise over 30 min. After the addition was complete, the reaction mixture was stirred overnight at 353 K. It was then quenched with 200 ml of water and extracted with 1-butanol (5 × 20 ml), the extract was then washed with water (2 ×10 ml). Evaporation of solvents from the extract on a rotary evaporator and recrystallization from isopropyl alcohol gave 3.83 g (87%) of the product as colourless crystals. ^1^H NMR (CDCI_3_), δ, ppm: 1.24 (*t*, 4H, γ-CH_2_, *J* = 7 Hz), 1.79 (*q*, 4H, β-CH_2_, *J* = 7 Hz), 4.06 (*t*, 4H, α-CH_2_, *J* = 7 Hz), 7.83 (*s*, 2H, H^3^-Tr), 8.08 (*s*, 2H, H^5^-Tr). ^13^C NMR (CDCI_3_), δ, ppm: 25.6 (γ-CH_2_), 29.3 (β-CH_2_), 49.2 (α –CH_2_), 142.7 (Tr-C^3^), 151.6 (Tr-C^5^).

## Refinement   

Crystal data, data collection and structure refinement details are summarized in Table 2 ▸. H atoms were refined as riding atoms (C—H = 0.97 Å with *U*_iso_(H) = 1.5*U*_eq_(C) for methyl H atoms and C—H = 0.93 Å 1.2*U*_eq_(C) for all others. Methyl H atoms were refined as rotating groups.

## Supplementary Material

Crystal structure: contains datablock(s) I. DOI: 10.1107/S2056989017017224/eb2003sup1.cif

Structure factors: contains datablock(s) I. DOI: 10.1107/S2056989017017224/eb2003Isup2.hkl

Supplementary figures S1-S6. DOI: 10.1107/S2056989017017224/eb2003sup3.pdf

CCDC reference: 1588505

Additional supporting information:  crystallographic information; 3D view; checkCIF report

## Figures and Tables

**Table 1:** Hydrogen-bond geometry (Å, °) *Cg* is the centroid of the C24–C26/C24^1^–C26^i^ ring.

*D*—H⋯*A*	*D*—H	H⋯*A*	*D*⋯*A*	*D*—H⋯*A*
C36^ii^—H36^ii^⋯*Cg*	0.93	3.07	3.95	149

Symmetry codes: (i) 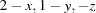; (ii) 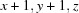.

**Table 2:** Experimental details

Crystal data	
Chemical formula	[Zn(C_8_H_4_O_4_)(C_10_H_16_N_6_)]·C_3_H_7_NO
*M*_r_	522.86
Crystal system, space group	Triclinic, *P* 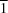
Temperature (K)	298
*a*, *b*, *c* (Å)	9.7803 (6), 10.4481 (5), 13.3708 (8)
α, β, γ (°)	101.438 (2), 101.015 (2), 109.073 (2)
*V* (Å^3^)	1216.41 (12)
*Z*	2
Radiation type	Mo *K*α
μ (mm^−1^)	1.06
Crystal size (mm)	0.1 × 0.05 × 0.02

Data collection	
Diffractometer	Bruker APEXII CCD
Absorption correction	Multi-scan (*SADABS*; Bruker, 2012 ▸)
*T*_min_, *T*_max_	0.665, 0.745
No. of measured, independent and observed [*I* > 2σ(*I*)] reflections	12157, 4293, 2999
*R*_int_	0.049
(sin θ/λ)_max_ (Å^−1^)	0.595

Refinement	
*R*[*F* ^2^ > 2σ(*F* ^2^)], *wR*(*F* ^2^), *S*	0.047, 0.118, 1.00
No. of reflections	4293
No. of parameters	309
H-atom treatment	H-atom parameters constrained
Δρ_max_, Δρ_min_ (e Å^−3^)	0.60, −0.33

Computer programs: *APEX2* and *SAINT* (Bruker, 2012 ▸), *SHELXT* (Sheldrick, 2015*a* ▸), *SHELXL2014* (Sheldrick, 2015*b* ▸) and *OLEX2* (Dolomanov *et al.*, 2009 ▸).

## supplementary crystallographic information

### Crystal data

**Table:**

[Zn(C_8_H_4_O_4_)(C_10_H_16_N_6_)]·C_3_H_7_NO	*Z* = 2
*M**_r_* = 522.86	*F*(000) = 544
Triclinic, *P*1	*D*_x_ = 1.428 Mg m^−^^3^
*a* = 9.7803 (6) Å	Mo *K*α radiation, λ = 0.71073 Å
*b* = 10.4481 (5) Å	Cell parameters from 2516 reflections
*c* = 13.3708 (8) Å	θ = 2.3–22.4°
α = 101.438 (2)°	µ = 1.06 mm^−^^1^
β = 101.015 (2)°	*T* = 298 K
γ = 109.073 (2)°	Plate, colourless
*V* = 1216.41 (12) Å^3^	0.1 × 0.05 × 0.02 mm

### Data collection

**Table:**

Bruker APEXII CCD diffractometer	2999 reflections with *I* > 2σ(*I*)
φ and ω scans	*R*_int_ = 0.049
Absorption correction: multi-scan (SADABS; Bruker, 2012)	θ_max_ = 25.0°, θ_min_ = 2.2°
*T*_min_ = 0.665, *T*_max_ = 0.745	*h* = −11→11
12157 measured reflections	*k* = −12→9
4293 independent reflections	*l* = −15→15

### Refinement

**Table:**

Refinement on *F*^2^	0 restraints
Least-squares matrix: full	Hydrogen site location: inferred from neighbouring sites
*R*[*F*^2^ > 2σ(*F*^2^)] = 0.047	H-atom parameters constrained
*wR*(*F*^2^) = 0.118	*w* = 1/[σ^2^(*F*_o_^2^) + (0.062*P*)^2^] where *P* = (*F*_o_^2^ + 2*F*_c_^2^)/3
*S* = 1.00	(Δ/σ)_max_ < 0.001
4293 reflections	Δρ_max_ = 0.60 e Å^−^^3^
309 parameters	Δρ_min_ = −0.33 e Å^−^^3^

### Special details

**Table:**

Geometry. All esds (except the esd in the dihedral angle between two l.s. planes) are estimated using the full covariance matrix. The cell esds are taken into account individually in the estimation of esds in distances, angles and torsion angles; correlations between esds in cell parameters are only used when they are defined by crystal symmetry. An approximate (isotropic) treatment of cell esds is used for estimating esds involving l.s. planes.

### Fractional atomic coordinates and isotropic or equivalent isotropic displacement parameters (Å^2^)

**Table:**

	*x*	*y*	*z*	*U*_iso_*/*U*_eq_	
Zn1	0.55113 (4)	0.42735 (4)	0.19802 (3)	0.04413 (17)	
O21	0.6685 (3)	0.4028 (3)	0.0983 (2)	0.0588 (7)	
O22	0.8610 (3)	0.5441 (3)	0.2351 (2)	0.0751 (9)	
O31	0.3399 (3)	0.3182 (3)	0.1154 (3)	0.0745 (9)	
O32	0.3836 (4)	0.1533 (3)	0.1758 (3)	0.0911 (11)	
N111	0.6118 (3)	0.4320 (3)	0.3513 (2)	0.0476 (7)	
N113	0.6160 (4)	0.3855 (4)	0.5075 (3)	0.0749 (11)	
N114	0.7283 (4)	0.5081 (3)	0.5193 (3)	0.0586 (9)	
N121	1.4483 (3)	1.3736 (3)	0.7898 (2)	0.0464 (7)	
N123	1.4969 (4)	1.1793 (4)	0.7385 (3)	0.0701 (10)	
N124	1.4569 (3)	1.1841 (3)	0.8295 (3)	0.0504 (8)	
C23	0.8065 (4)	0.4779 (4)	0.1401 (3)	0.0486 (9)	
C24	0.9064 (4)	0.4885 (3)	0.0678 (3)	0.0436 (9)	
C25	1.0606 (4)	0.5593 (4)	0.1062 (3)	0.0654 (12)	
H25	1.1032	0.6004	0.1788	0.078*	
C26	1.1517 (4)	0.5703 (4)	0.0403 (3)	0.0637 (11)	
H26	1.2551	0.6186	0.0688	0.076*	
C33	0.3002 (4)	0.1957 (4)	0.1235 (3)	0.0540 (10)	
C34	0.1434 (4)	0.0945 (4)	0.0602 (3)	0.0473 (9)	
C35	0.0949 (4)	−0.0457 (4)	0.0597 (3)	0.0544 (10)	
H35	0.1584	−0.0773	0.1002	0.065*	
C36	−0.0471 (4)	−0.1389 (4)	−0.0003 (3)	0.0547 (10)	
H36	−0.0781	−0.2331	−0.0003	0.066*	
C112	0.5502 (5)	0.3442 (4)	0.4054 (4)	0.0674 (12)	
H112	0.4670	0.2605	0.3729	0.081*	
C115	0.7231 (5)	0.5337 (4)	0.4267 (3)	0.0596 (11)	
H115	0.7894	0.6132	0.4158	0.071*	
C122	1.4891 (5)	1.2946 (4)	0.7177 (3)	0.0658 (12)	
H122	1.5100	1.3199	0.6577	0.079*	
C125	1.4289 (4)	1.2994 (4)	0.8595 (3)	0.0496 (9)	
H125	1.3999	1.3247	0.9204	0.060*	
C131	0.8254 (6)	0.5943 (5)	0.6262 (3)	0.0807 (14)	
H13A	0.8279	0.5326	0.6714	0.097*	
H13B	0.7810	0.6584	0.6554	0.097*	
C132	0.9823 (5)	0.6778 (4)	0.6292 (3)	0.0662 (12)	
H13C	0.9812	0.7343	0.5797	0.079*	
H13D	1.0317	0.6146	0.6076	0.079*	
C133	1.0684 (5)	0.7730 (4)	0.7397 (3)	0.0656 (12)	
H13E	1.0744	0.7146	0.7871	0.079*	
H13F	1.0118	0.8284	0.7629	0.079*	
C134	1.2262 (5)	0.8728 (4)	0.7510 (3)	0.0628 (11)	
H13G	1.2858	0.8186	0.7319	0.075*	
H13H	1.2222	0.9302	0.7025	0.075*	
C135	1.3004 (5)	0.9675 (4)	0.8631 (3)	0.0636 (12)	
H13I	1.2358	1.0160	0.8823	0.076*	
H13J	1.3056	0.9083	0.9100	0.076*	
C136	1.4545 (5)	1.0756 (4)	0.8842 (3)	0.0654 (12)	
H13K	1.4953	1.1204	0.9600	0.078*	
H13L	1.5191	1.0290	0.8617	0.078*	
O1S	1.2093 (8)	1.1555 (6)	0.4821 (6)	0.225 (4)	
N3S	1.1710 (7)	0.9340 (5)	0.4102 (4)	0.1045 (16)	
C2S	1.2272 (11)	1.0486 (9)	0.4748 (8)	0.179 (4)	
H2S	1.3017	1.0532	0.5318	0.215*	
C4S	1.1971 (10)	0.8107 (8)	0.4102 (7)	0.175 (4)	
H4SA	1.2829	0.8303	0.4680	0.263*	
H4SB	1.2159	0.7757	0.3443	0.263*	
H4SC	1.1104	0.7411	0.4181	0.263*	
C5S	1.0403 (15)	0.9099 (11)	0.3279 (8)	0.261 (7)	
H5SA	0.9604	0.9146	0.3586	0.392*	
H5SB	1.0094	0.8183	0.2790	0.392*	
H5SC	1.0637	0.9807	0.2909	0.392*	

### Atomic displacement parameters (Å^2^)

**Table:**

	*U*^11^	*U*^22^	*U*^33^	*U*^12^	*U*^13^	*U*^23^
Zn1	0.0345 (2)	0.0472 (3)	0.0430 (3)	0.00801 (18)	0.01047 (19)	0.00923 (18)
O21	0.0449 (16)	0.0665 (17)	0.0534 (18)	0.0082 (13)	0.0217 (14)	0.0061 (13)
O22	0.0524 (18)	0.100 (2)	0.052 (2)	0.0115 (16)	0.0196 (16)	0.0013 (17)
O31	0.0462 (17)	0.0557 (18)	0.097 (3)	0.0006 (13)	0.0069 (16)	0.0137 (16)
O32	0.061 (2)	0.070 (2)	0.104 (3)	0.0154 (16)	−0.0199 (19)	−0.0029 (18)
N111	0.0452 (18)	0.0493 (17)	0.0420 (19)	0.0111 (14)	0.0116 (16)	0.0114 (15)
N113	0.070 (3)	0.078 (2)	0.053 (3)	0.000 (2)	0.008 (2)	0.0255 (19)
N114	0.055 (2)	0.061 (2)	0.045 (2)	0.0058 (17)	0.0104 (17)	0.0148 (16)
N121	0.0449 (18)	0.0446 (17)	0.0431 (19)	0.0117 (14)	0.0118 (15)	0.0077 (15)
N123	0.093 (3)	0.069 (2)	0.063 (3)	0.041 (2)	0.037 (2)	0.0171 (19)
N124	0.0488 (19)	0.0504 (19)	0.046 (2)	0.0174 (15)	0.0054 (16)	0.0093 (15)
C23	0.045 (2)	0.055 (2)	0.051 (3)	0.0197 (19)	0.021 (2)	0.016 (2)
C24	0.038 (2)	0.046 (2)	0.043 (2)	0.0121 (16)	0.0134 (18)	0.0102 (17)
C25	0.042 (2)	0.092 (3)	0.040 (3)	0.011 (2)	0.008 (2)	−0.001 (2)
C26	0.032 (2)	0.093 (3)	0.048 (3)	0.008 (2)	0.010 (2)	0.006 (2)
C33	0.039 (2)	0.056 (3)	0.060 (3)	0.017 (2)	0.017 (2)	0.002 (2)
C34	0.036 (2)	0.045 (2)	0.054 (3)	0.0101 (16)	0.0138 (18)	0.0059 (17)
C35	0.041 (2)	0.051 (2)	0.065 (3)	0.0149 (18)	0.008 (2)	0.0133 (19)
C36	0.048 (2)	0.042 (2)	0.068 (3)	0.0121 (18)	0.014 (2)	0.0144 (19)
C112	0.061 (3)	0.063 (3)	0.058 (3)	−0.001 (2)	0.009 (2)	0.022 (2)
C115	0.061 (3)	0.059 (2)	0.048 (3)	0.007 (2)	0.017 (2)	0.017 (2)
C122	0.072 (3)	0.071 (3)	0.054 (3)	0.024 (2)	0.026 (2)	0.015 (2)
C125	0.044 (2)	0.053 (2)	0.047 (2)	0.0171 (18)	0.0098 (18)	0.0097 (19)
C131	0.087 (4)	0.083 (3)	0.044 (3)	0.006 (3)	0.008 (3)	0.010 (2)
C132	0.054 (3)	0.074 (3)	0.056 (3)	0.022 (2)	0.003 (2)	0.001 (2)
C133	0.071 (3)	0.057 (2)	0.052 (3)	0.016 (2)	0.002 (2)	0.008 (2)
C134	0.060 (3)	0.065 (3)	0.055 (3)	0.026 (2)	0.005 (2)	0.006 (2)
C135	0.076 (3)	0.055 (2)	0.049 (3)	0.021 (2)	0.001 (2)	0.0133 (19)
C136	0.077 (3)	0.057 (2)	0.054 (3)	0.028 (2)	−0.003 (2)	0.012 (2)
O1S	0.253 (8)	0.094 (4)	0.257 (8)	0.083 (4)	−0.056 (6)	−0.012 (4)
N3S	0.146 (5)	0.079 (3)	0.084 (4)	0.049 (3)	0.020 (3)	0.013 (3)
C2S	0.214 (10)	0.096 (6)	0.187 (9)	0.047 (6)	0.008 (8)	0.017 (6)
C4S	0.199 (9)	0.149 (7)	0.189 (9)	0.099 (7)	0.052 (7)	0.017 (6)
C5S	0.345 (17)	0.179 (10)	0.186 (10)	0.104 (10)	−0.075 (12)	0.026 (8)

### Geometric parameters (Å, º)

**Table:**

Zn1—O21	1.950 (3)	C36—H36	0.9300
Zn1—O31	1.969 (3)	C112—H112	0.9300
Zn1—N111	2.008 (3)	C115—H115	0.9300
Zn1—N121^i^	2.052 (3)	C122—H122	0.9300
O21—C23	1.263 (4)	C125—H125	0.9300
O22—C23	1.236 (4)	C131—H13A	0.9700
O31—C33	1.244 (5)	C131—H13B	0.9700
O32—C33	1.222 (5)	C131—C132	1.485 (6)
N111—C112	1.340 (5)	C132—H13C	0.9700
N111—C115	1.315 (5)	C132—H13D	0.9700
N113—N114	1.344 (4)	C132—C133	1.509 (5)
N113—C112	1.309 (5)	C133—H13E	0.9700
N114—C115	1.314 (5)	C133—H13F	0.9700
N114—C131	1.472 (5)	C133—C134	1.513 (6)
N121—Zn1^i^	2.052 (3)	C134—H13G	0.9700
N121—C122	1.348 (5)	C134—H13H	0.9700
N121—C125	1.327 (5)	C134—C135	1.510 (5)
N123—N124	1.343 (4)	C135—H13I	0.9700
N123—C122	1.311 (5)	C135—H13J	0.9700
N124—C125	1.324 (4)	C135—C136	1.492 (6)
N124—C136	1.462 (5)	C136—H13K	0.9700
C23—C24	1.495 (5)	C136—H13L	0.9700
C24—C25	1.382 (5)	O1S—C2S	1.174 (8)
C24—C26^ii^	1.376 (5)	N3S—C2S	1.208 (9)
C25—H25	0.9300	N3S—C4S	1.393 (8)
C25—C26	1.363 (5)	N3S—C5S	1.432 (10)
C26—C24^ii^	1.376 (5)	C2S—H2S	0.9300
C26—H26	0.9300	C4S—H4SA	0.9600
C33—C34	1.508 (5)	C4S—H4SB	0.9600
C34—C35	1.383 (5)	C4S—H4SC	0.9600
C34—C36^iii^	1.374 (5)	C5S—H5SA	0.9600
C35—H35	0.9300	C5S—H5SB	0.9600
C35—C36	1.379 (5)	C5S—H5SC	0.9600
C36—C34^iii^	1.374 (5)		
			
O21—Zn1—O31	105.00 (12)	N124—C125—N121	110.0 (4)
O21—Zn1—N111	124.42 (12)	N124—C125—H125	125.0
O21—Zn1—N121^i^	104.47 (12)	N114—C131—H13A	108.7
O31—Zn1—N111	118.44 (13)	N114—C131—H13B	108.7
O31—Zn1—N121^i^	98.53 (12)	N114—C131—C132	114.2 (4)
N111—Zn1—N121^i^	101.66 (12)	H13A—C131—H13B	107.6
C23—O21—Zn1	110.9 (2)	C132—C131—H13A	108.7
C33—O31—Zn1	110.0 (3)	C132—C131—H13B	108.7
C112—N111—Zn1	131.6 (3)	C131—C132—H13C	109.6
C115—N111—Zn1	126.1 (3)	C131—C132—H13D	109.6
C115—N111—C112	102.1 (3)	C131—C132—C133	110.3 (4)
C112—N113—N114	102.6 (3)	H13C—C132—H13D	108.1
N113—N114—C131	119.8 (3)	C133—C132—H13C	109.6
C115—N114—N113	109.4 (3)	C133—C132—H13D	109.6
C115—N114—C131	130.7 (4)	C132—C133—H13E	108.5
C122—N121—Zn1^i^	128.6 (3)	C132—C133—H13F	108.5
C125—N121—Zn1^i^	128.2 (3)	C132—C133—C134	115.3 (4)
C125—N121—C122	102.5 (3)	H13E—C133—H13F	107.5
C122—N123—N124	102.6 (3)	C134—C133—H13E	108.5
N123—N124—C136	121.4 (3)	C134—C133—H13F	108.5
C125—N124—N123	110.2 (3)	C133—C134—H13G	109.4
C125—N124—C136	128.4 (4)	C133—C134—H13H	109.4
O21—C23—C24	116.7 (3)	H13G—C134—H13H	108.0
O22—C23—O21	123.9 (4)	C135—C134—C133	111.3 (4)
O22—C23—C24	119.4 (3)	C135—C134—H13G	109.4
C25—C24—C23	121.5 (4)	C135—C134—H13H	109.4
C26^ii^—C24—C23	121.3 (3)	C134—C135—H13I	108.1
C26^ii^—C24—C25	117.1 (3)	C134—C135—H13J	108.1
C24—C25—H25	119.2	H13I—C135—H13J	107.3
C26—C25—C24	121.7 (4)	C136—C135—C134	116.8 (4)
C26—C25—H25	119.2	C136—C135—H13I	108.1
C24^ii^—C26—H26	119.4	C136—C135—H13J	108.1
C25—C26—C24^ii^	121.2 (4)	N124—C136—C135	113.0 (3)
C25—C26—H26	119.4	N124—C136—H13K	109.0
O31—C33—C34	117.3 (4)	N124—C136—H13L	109.0
O32—C33—O31	123.2 (4)	C135—C136—H13K	109.0
O32—C33—C34	119.4 (4)	C135—C136—H13L	109.0
C35—C34—C33	120.5 (3)	H13K—C136—H13L	107.8
C36^iii^—C34—C33	120.8 (3)	C2S—N3S—C4S	130.5 (8)
C36^iii^—C34—C35	118.7 (3)	C2S—N3S—C5S	116.7 (7)
C34—C35—H35	119.8	C4S—N3S—C5S	112.0 (6)
C36—C35—C34	120.4 (3)	O1S—C2S—N3S	134.7 (10)
C36—C35—H35	119.8	O1S—C2S—H2S	112.7
C34^iii^—C36—C35	120.8 (3)	N3S—C2S—H2S	112.7
C34^iii^—C36—H36	119.6	N3S—C4S—H4SA	109.5
C35—C36—H36	119.6	N3S—C4S—H4SB	109.5
N111—C112—H112	122.6	N3S—C4S—H4SC	109.5
N113—C112—N111	114.8 (4)	H4SA—C4S—H4SB	109.5
N113—C112—H112	122.6	H4SA—C4S—H4SC	109.5
N111—C115—H115	124.4	H4SB—C4S—H4SC	109.5
N114—C115—N111	111.1 (4)	N3S—C5S—H5SA	109.5
N114—C115—H115	124.4	N3S—C5S—H5SB	109.5
N121—C122—H122	122.6	N3S—C5S—H5SC	109.5
N123—C122—N121	114.8 (4)	H5SA—C5S—H5SB	109.5
N123—C122—H122	122.6	H5SA—C5S—H5SC	109.5
N121—C125—H125	125.0	H5SB—C5S—H5SC	109.5
			
Zn1—O21—C23—O22	8.3 (5)	C24—C25—C26—C24^ii^	0.1 (7)
Zn1—O21—C23—C24	−170.1 (2)	C26^ii^—C24—C25—C26	−0.1 (7)
Zn1—O31—C33—O32	−0.1 (5)	C33—C34—C35—C36	−178.3 (4)
Zn1—O31—C33—C34	−176.8 (3)	C34—C35—C36—C34^iii^	−0.5 (7)
Zn1—N111—C112—N113	−176.0 (3)	C36^iii^—C34—C35—C36	0.5 (7)
Zn1—N111—C115—N114	176.7 (3)	C112—N111—C115—N114	0.3 (5)
Zn1^i^—N121—C122—N123	170.8 (3)	C112—N113—N114—C115	0.6 (5)
Zn1^i^—N121—C125—N124	−171.3 (2)	C112—N113—N114—C131	176.1 (4)
O21—C23—C24—C25	−175.2 (3)	C115—N111—C112—N113	0.0 (5)
O21—C23—C24—C26^ii^	5.7 (5)	C115—N114—C131—C132	−36.4 (7)
O22—C23—C24—C25	6.4 (5)	C122—N121—C125—N124	0.1 (4)
O22—C23—C24—C26^ii^	−172.7 (4)	C122—N123—N124—C125	−0.6 (4)
O31—C33—C34—C35	175.8 (4)	C122—N123—N124—C136	−178.1 (3)
O31—C33—C34—C36^iii^	−3.0 (6)	C125—N121—C122—N123	−0.5 (5)
O32—C33—C34—C35	−1.0 (6)	C125—N124—C136—C135	83.2 (5)
O32—C33—C34—C36^iii^	−179.8 (4)	C131—N114—C115—N111	−175.5 (4)
N113—N114—C115—N111	−0.6 (5)	C131—C132—C133—C134	−175.0 (4)
N113—N114—C131—C132	149.1 (4)	C132—C133—C134—C135	177.6 (3)
N114—N113—C112—N111	−0.4 (5)	C133—C134—C135—C136	−177.4 (3)
N114—C131—C132—C133	174.5 (4)	C134—C135—C136—N124	66.5 (5)
N123—N124—C125—N121	0.4 (4)	C136—N124—C125—N121	177.7 (3)
N123—N124—C136—C135	−99.8 (5)	C4S—N3S—C2S—O1S	−177.8 (11)
N124—N123—C122—N121	0.7 (5)	C5S—N3S—C2S—O1S	−9.0 (19)
C23—C24—C25—C26	−179.3 (4)		

Symmetry codes: (i) −*x*+2, −*y*+2, −*z*+1; (ii) −*x*+2, −*y*+1, −*z*; (iii) −*x*, −*y*, −*z*.

### Hydrogen-bond geometry (Å, º)

Cg is the centroid of the C24–C26/C24^i^–C26^i^ ring.

**Table:**

*D*—H···*A*	*D*—H	H···*A*	*D*···*A*	*D*—H···*A*
C36^iv^—H36^iv^···*Cg*	0.93	3.07	3.95	149

Symmetry code: (iv) *x*+1, *y*+1, *z*.
