# Supplementary material for: Crystal structure of a Zn complex with tereph­thalate and 1,6-bis­(1,2,4-triazol-1-yl)hexa­ne
Source: Acta Crystallogr E Crystallogr Commun. 2018 Jan 1;74(Pt 1):6–9. doi: 10.1107/S2056989017017224 (PMC5778475; doi:10.1107/S2056989017017224)
Supplement: Supplementary file 3 [file e-74-00006-sup3.pdf]

Supporting information

Crystal structure of Zn complex with terephthalate and 1,6-bis(1,2,4-triazol-1-yl)hexane, DMF solvate

by T.S. Sukhikh, E. Yu. Semitut and A. S. Potapov

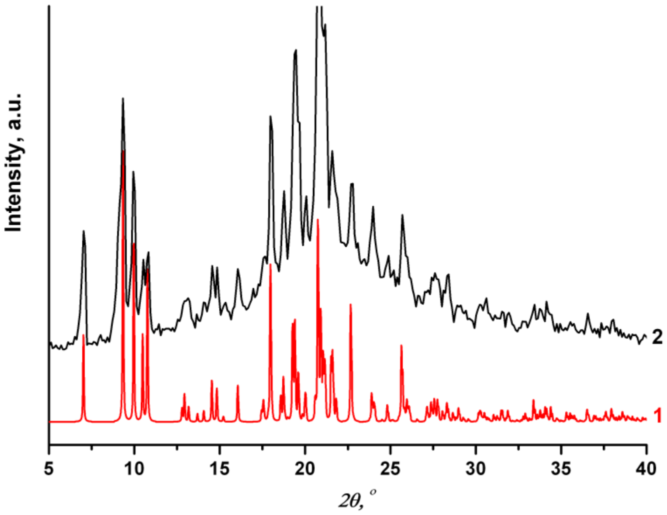

Figure S1. The XRD patterns of [Zn(btrh)(bdc)]·nDMF: 1 – simulated from single crystal data, 2 – polycrystalline sample.

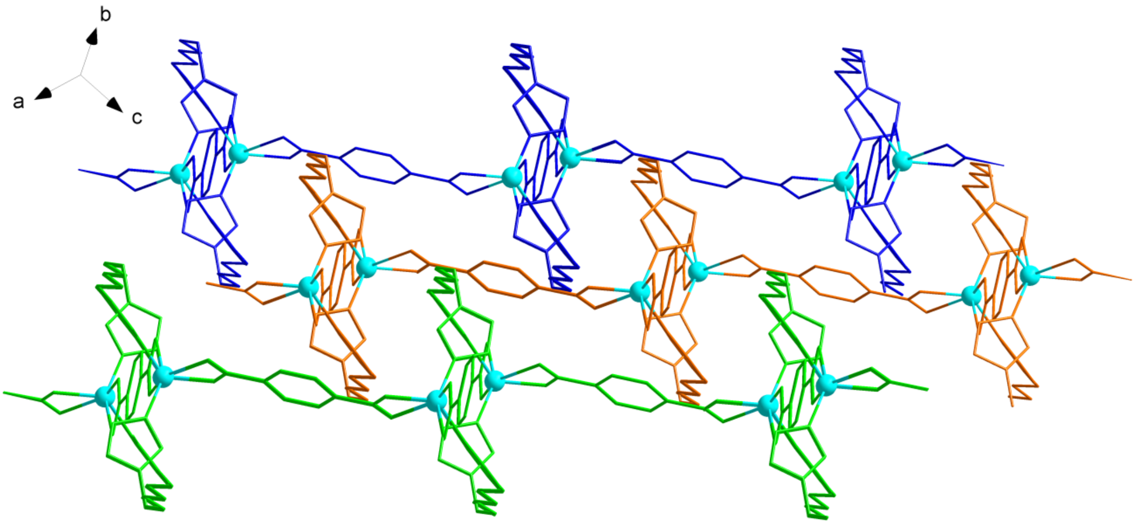

Figure S2. Relative arrangement of the layers of the coordination polymer. Different layers are colored with orange, green and blue. H atoms are not shown for clarity.

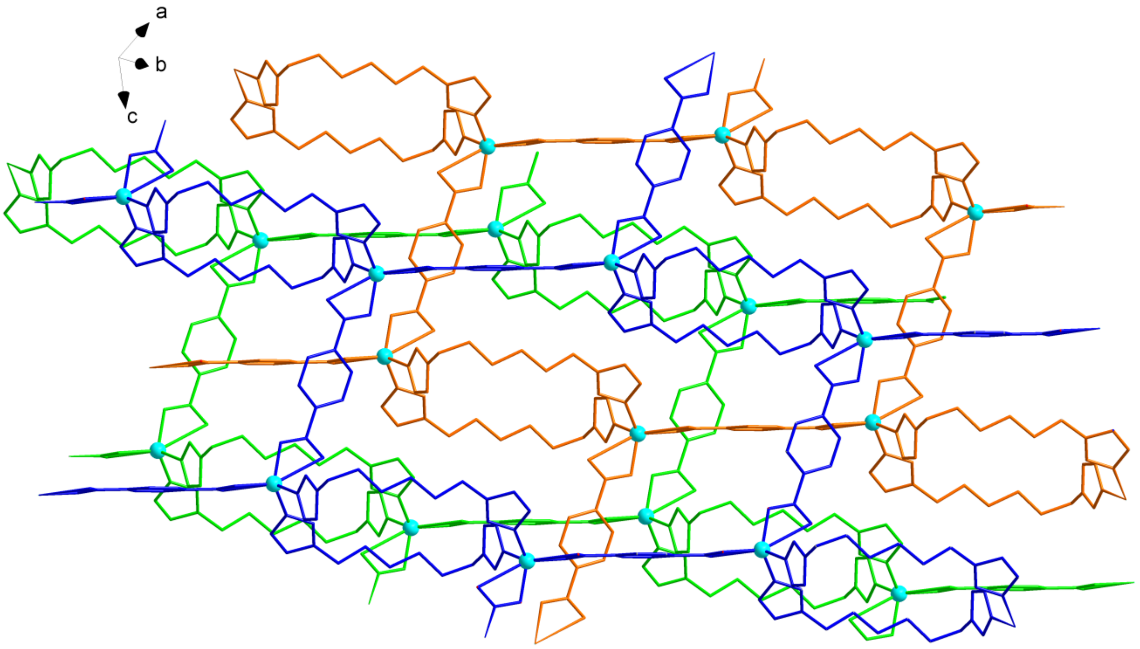

Figure S3. Relative arrangement of the layers of the coordination polymer. Different layers are colored with orange, green and blue. H atoms are not shown for clarity.

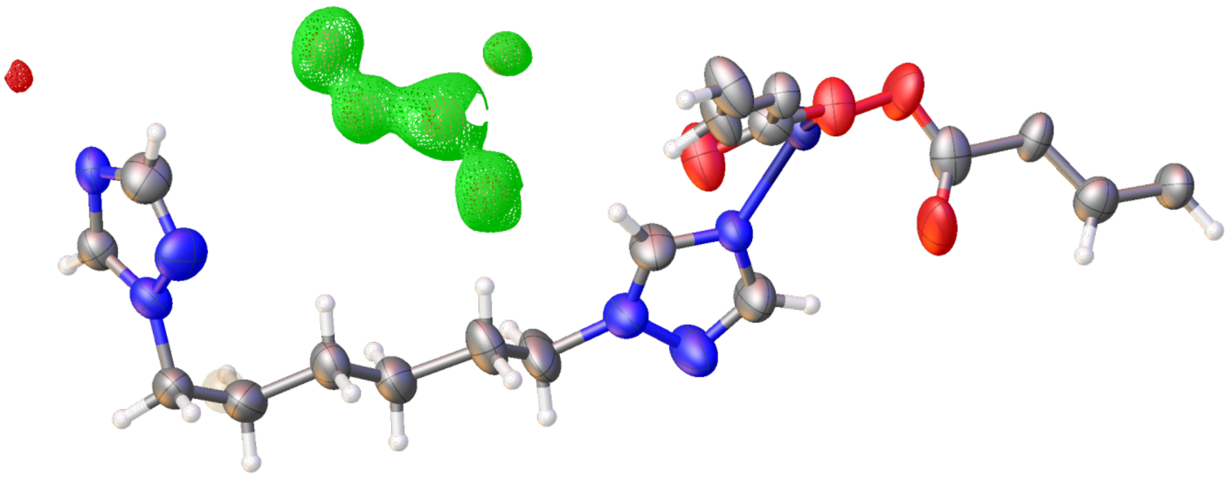

Figure S4. Residual electron density map for the structure [Zn(btrh)(bdc)] without solvent DMF: green – negative, red – positive.

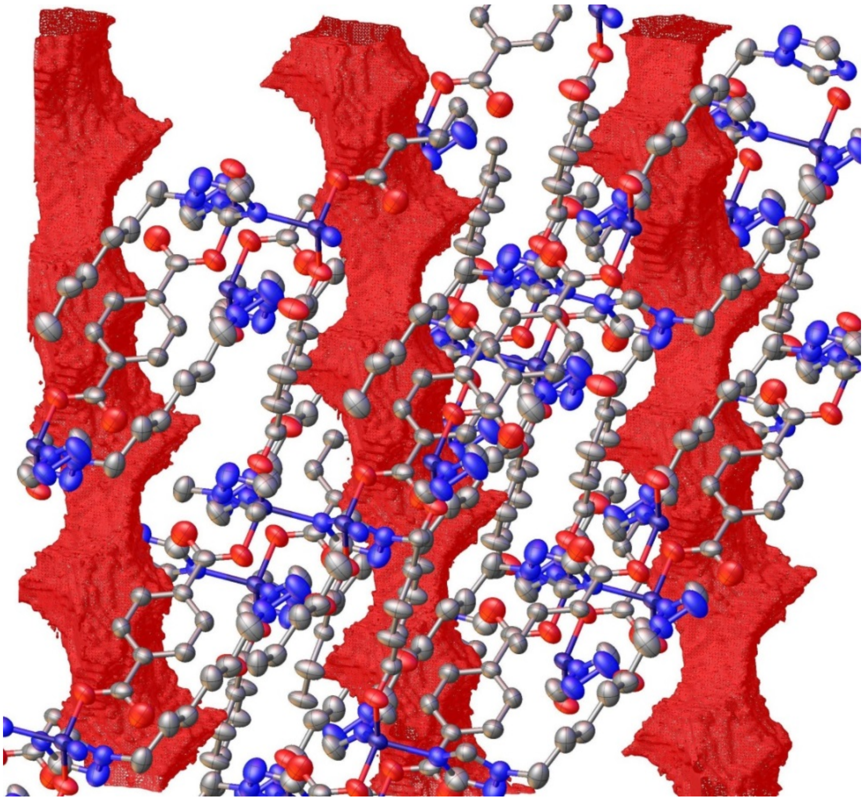

Figure S5. Representation of channel voids in the structure where DMF solvent molecules are located. H atoms and solvent molecules are not shown.

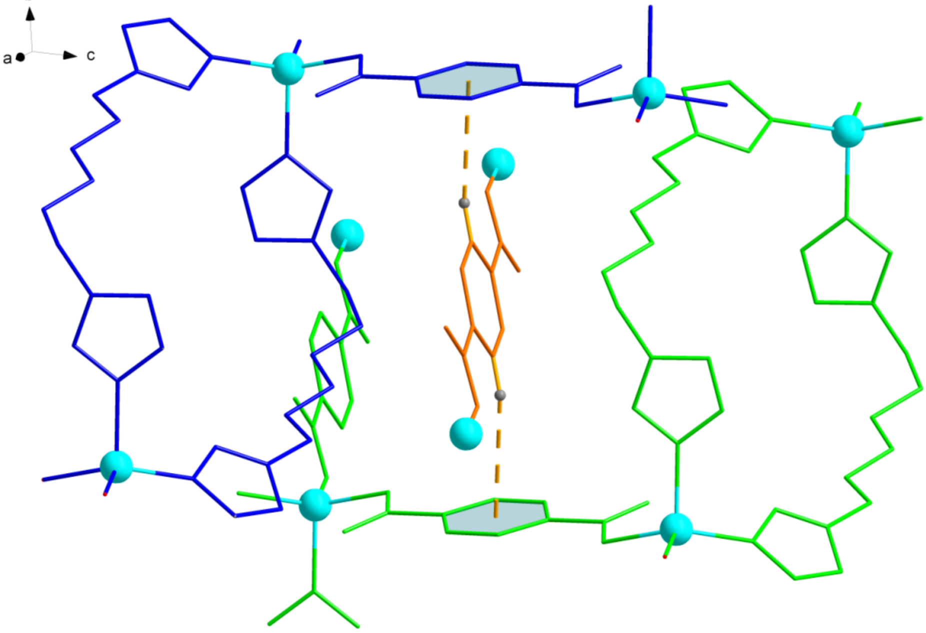

Fig. S6. Representation of fragment of the structure, showing CH···π contacts (marked dashed). Different layers are colored with orange, green and blue. H atoms are not shown for clarity.
